# Influence of Mg Content on Microstructure Coarsening, Molten Pool Size, and Hardness of Laser Remelted Al(-*x*)–Mg–Sc Alloys

**DOI:** 10.1021/acsomega.4c06126

**Published:** 2024-08-28

**Authors:** Anderson Thadeu Nunes, Rudimar Riva, Aline Gonçalves Capella, Amauri Garcia, José Eduardo Spinelli

**Affiliations:** † Graduate Program in Materials Science and Engineering, Federal University of São Carlos, 13565-905 São Carlos, São Paulo, Brazil; ‡ Federal University of São Paulo - ICT, Rua Talim, 330 − Vila Nair, 12231-280 São José dos Campos, São Paulo, Brazil; § Department of Manufacturing and Materials Engineering, University of Campinas, 13083-860 Campinas, São Paulo Brazil; ∥ Department of Materials Engineering, Federal University of São Carlos UFSCar, 13565-905 São Carlos, São Paulo Brazil

## Abstract

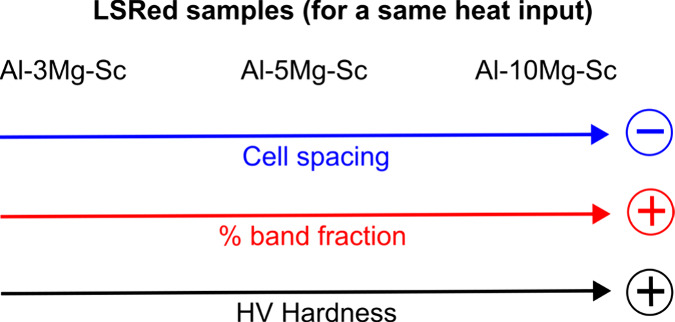

Investigations leading to high-quality surfaces and optimized properties in laser remelted Al–Mg–Sc alloys remain scarce. Laser surface remelting (LSR) has been used for the final treatment of these alloys processed through additive manufacturing. However, the direct microstructure responses of the treated cast surfaces have not yet been investigated. In the present research, Al-3, 5, and 10 wt %–Mg-0.1 wt %–Sc alloys plates were processed using LSR to study the effects of local melting and rapid solidification. The morphology of the *α-Al* phase, microstructure coarsening, and hardness were mapped from the bottom to the top of the molten pools, varying with local Mg content and laser heat input (2.5 J/mm, 5 J/mm, and 10 J/mm). This study aimed to create a comprehensive map of the microstructures, hardness, and molten pool sizes under various conditions. The findings may help to optimize these alloys through understanding laser processing parameters. Methods used included CALPHAD computations, optical microscopy, SEM, EDS, image analysis, hardness tests, and heat flow models. The results obtained showed α-Al cell growth with bands in all alloys with hardness changes correlating with cell spacing and heat input. Higher Mg content resulted in more refined cells and a higher fraction of bands. Increased Mg content decreased the thermal diffusivity and enthalpy of melting, enlarging the molten pool size. Hardness increased with decreasing heat input and higher Mg content in the tested alloys, especially in the Al-10 wt %–Mg-0.1 wt %–Sc alloy as the heat input was varied.

## 1 Introduction

A thin surface layer of a material is melted and subsequently solidified by using a laser treatment in the manufacturing process known as laser surface remelting (LSR). Through this process, both the hardness and wear resistance of the treated surface can be enhanced, resulting in increased durability. LSR can also improve the alloy surface finish, giving it a smoother aspect. By relieving residual stresses, LSR can improve mechanical properties and decrease the chance that a component that has undergone such a treatment fails in service. LSR can provide exact control over the treatment area and depth. Therefore, LSR is important for enhancing the quality, durability, and performance of alloys in a wide range of industries.^1,2^ Microhardness refers to the hardness of materials when subjected to lower loads, and it is often used to estimate the overall mechanical strength.^1,2^ Typically, microhardness increases as the alloy microstructure becomes finer. This property is crucial for understanding how the LSR affects the surface microstructure. Additionally, the wear resistance depends on both the surface structure and hardness. Finer grains lead to improved wear resistance due to the increased hardness. Therefore, LSR is a valuable technique for enhancing both the hardness and the wear resistance of a given alloy.

Surface defects and features, such as surface roughness, pores, and coarse grains, can adversely influence the mechanical properties. Pores act as stress concentrators. When a load is applied, the stress around the pores is significantly higher than that in the surrounding material, which can lead to crack nucleation and subsequent failure.^3,4^ Moreover, coarse grains result in fewer grain boundaries per unit volume when compared to fine grains. With fewer barriers, dislocations can move more easily, resulting in a lower mechanical strength. Increase in both porosity fraction and coarse grains can result in lower ultimate tensile strength, ductility, and fatigue life.^3^ Surface voids typically decrease the alloy tensile strength.^4^ The surface defects mentioned earlier can have an adverse impact on the fatigue life properties of the alloy.^5,6^ There are several surface finishing techniques to reduce surface defects.^3^ Most of them are outdated or have limitations on their use. LSR offers several key benefits over traditional surface heat treatments. It allows for precise energy control, enabling the selective treatment of specific areas, thereby reducing the risk of damage to adjacent areas. LSR produces a refined microstructure with fine grains, enhancing mechanical properties such as hardness and wear resistance. The rapid cooling rates of LSR can create unique microstructures that cannot be achieved with conventional methods. Additionally, LSR minimizes thermal distortion and residual stresses and reduces processing time. It is easily customizable and energy-efficient, focusing energy directly on the surface layer. Overall, LSR is a versatile, accurate, and efficient technique for improving surface properties, making it a valuable alternative to traditional methods.^1,2^

Therefore, the requirement exists for the development of an efficient technique capable of meeting all of the prerequisites of an ideal one. LSR treatment steps in to bridge this existing gap. It stands out as the most widely employed approach for enhancing surface quality and optimizing microstructure.^7,8^

LSR offers the benefit of targeting specific surfaces,^9^ which is sufficient to enhance their properties and performance, without the need for treating/melting the entire material, as is the case with other heat treatment methods. Typically, there are five primary laser types: gas lasers, solid-state lasers, fiber lasers, liquid lasers, and semiconductor lasers.^10^

Al–Mg alloys exhibit significant potential for use in various industrial sectors, as indicated by previous studies.^11−14^ The introduction of Sc into these alloys, along with appropriate heat treatment, may unlock new opportunities.^15,16^ However, when it comes to LSR, there is a noticeable lack of information regarding Al–Mg alloys. There is also a shortage of knowledge regarding the behavior of various formed molten pool zones, especially regarding a range of Mg content levels in alloys. Therefore, understanding the formed LSR microstructures is a task of prime importance. The more influential parameters changing LSR microstructures are laser power, scanning velocity, and heat input.^17−20^ An optimized set of laser parameters must be applied to get the desired microstructures depending upon applications. The microstructure and hardness of an alloy can be enhanced by finding the right balance between the laser power and scanning velocity. Similarly, achieving an ideal combination of power and pulse frequency can boost the corrosion resistance and hardness. However, excessive exposure time and power can lead to uneven remelting. Furthermore, increasing the energy density results in coarser grains associated with an increase in residual compressive stresses.

The use of analytical models for predicting certain aspects of the LSR can be very beneficial. Two points are noteworthy, such as the size of the molten pool and the coarsening of the microstructure through dendritic/cellular spacing. In both cases, there has been limited testing for Al alloys and no studies exist for Al–Mg alloys.

The aforementioned process parameter choices (i.e., laser power and scanning velocity) significantly influence the morphology of the molten pool, including its depth and width. In order to expedite the process of selecting parameters and mapping out the LSR process, there is a strong demand for analytical or semianalytical methods as alternatives to time-consuming experimental and computational approaches. Consequently, scaling laws that consider thermophysical properties and dimensional analysis have emerged within the LSR community. For instance, Hann et al.^21^ introduced a relationship between normalized molten pool depth and normalized enthalpy. The former represents the ratio of the molten pool depth to the laser spot diameter, while the latter signifies the ratio of the deposited energy to the enthalpy of melting. Recently, Naderi and colleagues^22^ investigated the effectiveness of this model and its various adaptations on IN718, IN625, Ti6Al4 V, and SS316L alloys, without verifying its applicability to Al alloys.

Hann et al.^21^ proposed that the nondimensional depth of the weld (δ*) is related to the laser parameters and the alloy parameters by the expression:^1^
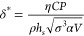
1where η = the absorptivity of the surface (= 0.3), *C* = a constant with no dimensions, *P* = laser power, ρ = density of the alloy, α = thermal diffusivity, *h*_*s*_ = enthalpy at melting, σ = half-width of the Gaussian beam at the surface, and *V* = laser speed. The molten pool depth can be expressed as *D* = δ* × 2σ.

Another analytical model of importance was proposed by Rosenthal.^23^ Except for the initial and final transients of laser treatments, heat flow in a substrate of sufficient length is steady with respect to the moving heat source.^24^ This assumption was employed to simplify the mathematical treatment of heat flow during welding and laser. Rosenthal’s analytical solutions are easy to use and have been highly valued by the industry. As such, the following expression for three-dimensional heat flow in a semi-infinite workpiece is well employed for calculating temperature distributions, and thermal gradients:
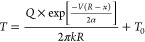
2where *T* = temperature, *T*_0_ = room temperature, *k* = thermal conductivity, *Q* = laser power transferred from heat source to substrate, *V* = laser scanning speed, α *=* alloy thermal diffusivity, and *R* = radial distance from origin, namely, (*x*^2^*+ y*^2^*+ z*^2^)^1/2^. Considering a heat transfer efficiency of 0.5, *Q* is the laser power input: *P* × 0.5.

Investigating laser-remelted Al–Mg–Sc alloys is crucial for advancing the understanding of how the formed microstructure, phase morphologies, and hardness are interconnected. Additionally, modeling the size of the laser-affected area will provide valuable insights for optimizing the LSR processes. This research could lead to significant improvements in the performance and reliability of Al–Mg–Sc used in various high-demand applications.

This research presents an innovative method to understand the effects of laser interactions on Al–Mg–Sc alloys, focusing on power and scanning speed, by using laser parameters and thermal/thermodynamic properties. The CALPHAD approach is employed to support the determination of these properties. Experimental results are analyzed, allowing the comprehension of micromorphological, microstructural coarsening, and hardness experimental evolutions as well as the validation of data obtained from molten pool size models against experimental evidence.

## 2 Materials and Methods

**Figure 1:**
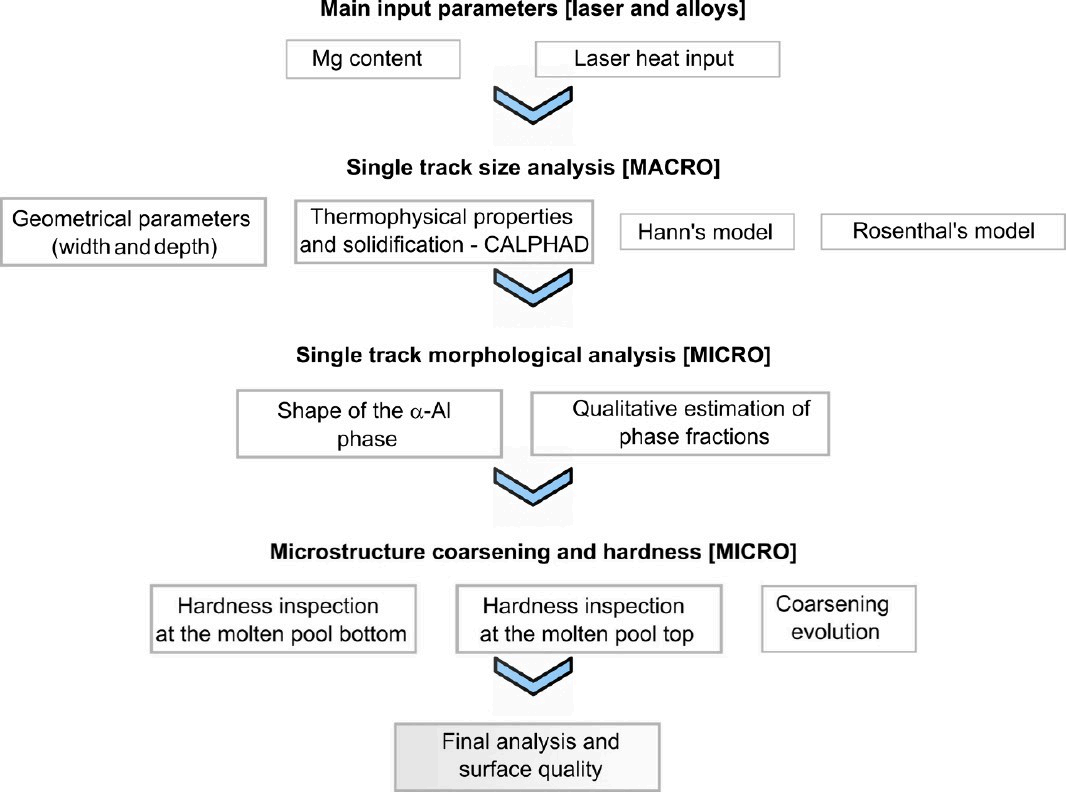
Flowchart showing the sequence of experimental activities for the analysis of the surface quality.

To systematically map alloys with varying Mg content processed under different laser heat inputs, a flowchart of methods was designed to facilitate the evaluation of key features, as shown in Figure 1. The first step involved selecting alloy Mg contents along with laser power and scanning speeds. The second step focused on determining the depth and width of the single laser tracks by comparing these measurements with theoretical predictions. The third and fourth steps were dedicated to microstructural analysis, emphasizing coarsening, morphologies, phase fractions, and hardness.

The Al-3, 5, and 10–Mg-0.1–Sc alloys were chosen for this study since they are considered lightweight alternatives for the aerospace industry. Plates of 25 × 21 mm, with thickness of 4 mm, were used. All plates were tested under their as-cast conditions. They were produced using a directional solidification apparatus to generate two distinct original microstructures: one corresponding to a solidification cooling rate of 2 K/s and the other of 5 K/s.^25,26^ The directionally solidified (DS) samples, used as substrates under the as-cast condition, were also characterized in terms of microstructural spacing and hardness with a view to compare as-cast (i.e., DS samples) and LSR processes. This methodology enabled the evaluation of all microstructural coarsening and hardness data of laser-treated samples in duplicate.

The surfaces of the substrates were prepared by sandblasting. A 500 W fiber laser manufactured by IPG, model YLR-500-MM-AC-Y14, was used to remelt the surface. The minimum spot diameter was 100 μm, and the wavelength was 1070 nm. The spot of the laser beam is due to the diameter of the optical fiber used in the experiments. During processing, the argon gas flow rate was used to protect the Al surface against oxidation. The shielding gas was directed over the irradiated area by using a rounded nozzle. The laser head was connected to a three-axis CNC motion system. Three tracks were produced in each substrate (duplicate) with a hatch spacing of 2 mm.

Table 1 shows the experimental conditions, where three groups of heat input values, 2.5 J/mm (Track 1), 5.0 J/mm (Track 2), and 10 J/mm (Track 3), were established to differentiate in 2× and 4× the energy supplied to the material during the process. The laser power (*P*) and laser beam speed (*V*) were set to maintain a constant heat input for each tested alloy. The focal distance was adjusted to achieve a spot size of 100 μm at the upper surface of the plate.

**Table 1:** Experimental Parameters to Produce the Single Tracks through LSR Processing

Alloy	Laser power, *P* (W)	Laser scanning speed, *V* (mm/s)	Heat input, HI (J/mm)
Al-3%–Mg-0.1%–Sc	250	25	Tracks 1–10
	250	50	Tracks 2–5
	250	100	Tracks 3–2.5
Al-5%–Mg-0.1%–Sc	250	25	Tracks 1–10
	250	50	Tracks 2–5
	250	100	Tracks 3–2.5
Al-10%–Mg-0.1%–Sc	250	25	Tracks 1–10
	250	50	Tracks 2–5
	250	100	Tracks 3–2.5

The investigation of the solidification sequence of Al–Mg–Sc alloys and the variation in the mass fraction of the phases formed were conducted as a function of temperature through thermodynamic simulations using the Thermo-Calc software version 2021a, which enables the CALPHAD (Computer Calculation of Phase Diagrams) method as its computational model,^27^ utilizing the TCAL 7 database. The use of this computational tool was also important as a source of thermodynamic and thermal properties for the theories for the depth and width of the laser molten pool. For the present CALPHAD calculations, a 0.2 wt % Fe content was considered for each alloy since Fe is a typical impurity in secondary Al ingots, like those used to generate the present alloys.

Optical microscopy, scanning electron microscopy (SEM), and energy dispersive X-ray spectroscopy (EDS) analyses were employed to investigate the phases and morphologies formed after the LSR process. To examine the microstructure/morphology features of the tracks, the samples were ground, polished, and etched using a solution of 1/3 HCl, 1/3 HNO_3_, 1/3 H_2_O, and 1/30 HF swabbed with cotton for 5 s. Images were captured with an optical microscope (Olympus BX41M-LED), and the cell spacing (*λc*) (or interphase spacing) at the bottom of the molten pool was determined using the intercept methodology.^28^ Microstructural details were assessed with scanning electron microscopy (SEM, Philips XL-30 FEG, Bruker).

Hardness measurements of the tracks were performed with a Vickers microhardness tester (Shimadzu HMV-G20ST) with a 10 gf load for 15 s at the bottom regions of the molten pool and 200 gf at the top regions. The ImageJ software^29^ was employed to measure the cell spacing at the bottom of the molten pool and to estimate cellular, intercellular, and band fractions. Considering that all images were acquired after chemical etching, only qualitative data is expected to be assessed with this image analysis. The procedure for estimating these fractions included (a) Selecting uniform-magnification optical images for all tracks of interest; (b) Removing nontrack regions from each image; (c) Adjusting the threshold; (d) Setting to B&W mode and measuring the track area within a range from 1 to 65,535; and (e) Fine-tuning the B&W range for accurate morphology representation and calculating the fractions. This process was repeated with three B&W ranges (130, 140, and 150 to 65,535) to obtain average and more representative values for all laser tracks.

## 3 Results and Discussion

### 3.1 Single Laser Tracks: Microstructures, Morphologies, and Molten Pool Sizes

**Figure 2:**
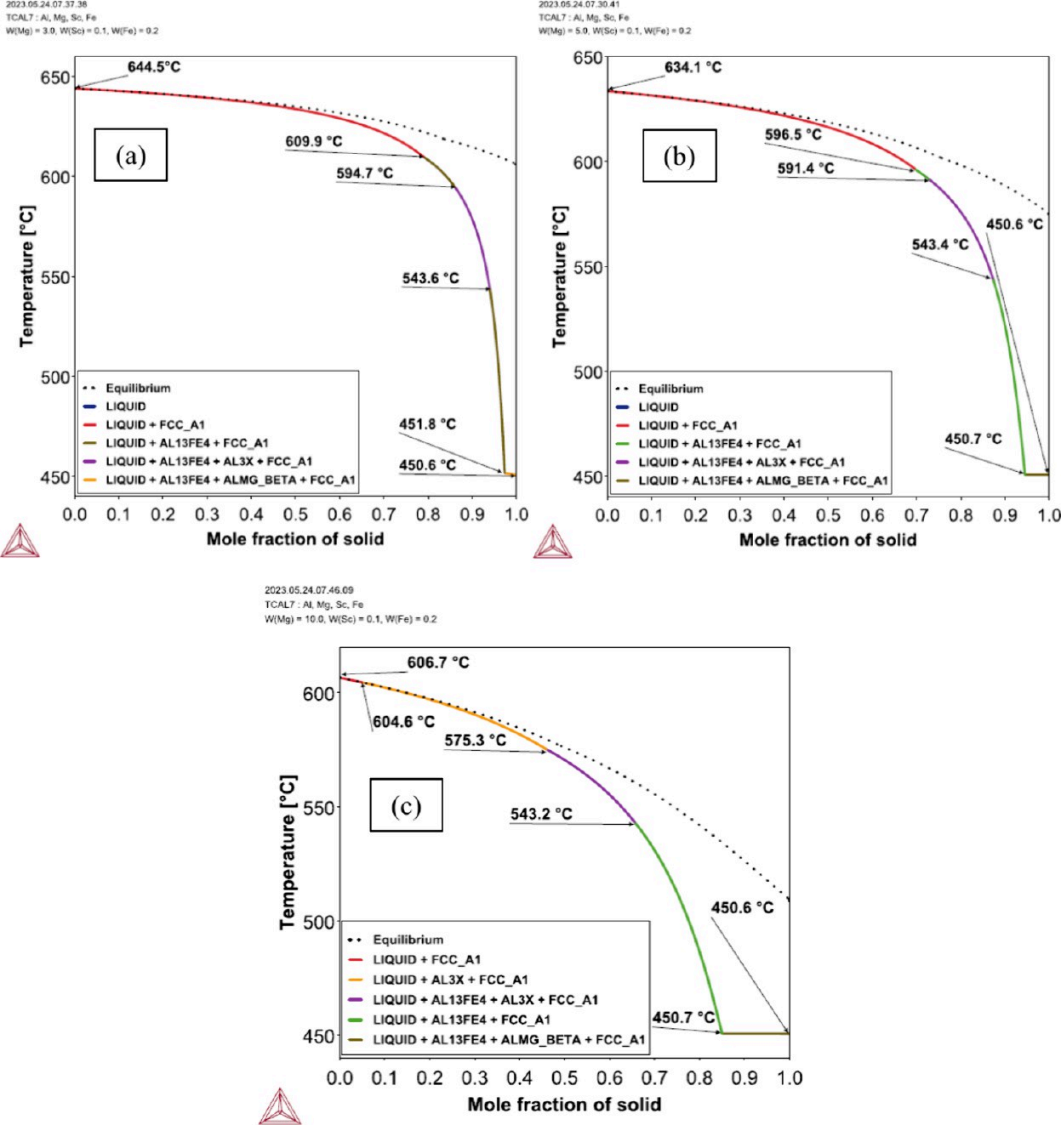
CALPHAD calculations from the TCAL 7 database for both equilibrium and nonequilibrium solidification of the (a) Al-3%–Mg-0.1%–Sc, (b) Al-5%–Mg-0.1%–Sc, and (c) Al-10%–Mg-0.1%–Sc alloys.

The CALPHAD method allowed for the calculation of the phase precipitation sequence during solidification of each alloy composition, resulting in either equilibrium or the Scheil profile, which assumes complete solute mixing in the liquid without diffusion in the solid. The Scheil model is considered suitable for predicting the phases formed under nonequilibrium conditions. Figure 2 shows the Scheil profiles for the Al-3%–Mg-0.1%–Sc, Al-5%–Mg-0.1%–Sc, and Al-10%–Mg-0.1%–Sc alloys obtained by thermodynamic calculations using the Thermo-Calc software and the TCAL7 database.

For the Al-3%–Mg-0.1%–Sc alloy, solidification begins at 644.5 °C with the formation of the *α-Al* phase, followed by the precipitation of the *Al*_13_*Fe*_4_ phase at 609.9 °C; the *Al*_3_*Sc* phase precipitates between 594.7 and 543.6 °C, and the *β-Al*_3_*Mg*_2_ phase is formed at 451.8 °C, while the remaining liquid (*L*) solidifies at 450.6 °C. The solidification range was approximately 194 K under nonequilibrium conditions, while under equilibrium conditions (dashed line in Figure 2(a)), it was about 40 K. The calculated solidification sequence was
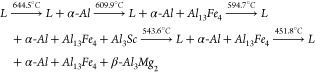
The intermetallic *Al*_3_*Sc* completed its formation at 543.6 °C.

The solidification path of the Al-5%–Mg-0.1%–Sc alloy in Figure 2(b) can be described as 
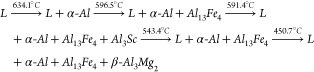
The intermetallic Al_3_Sc completed its formation at a temperature of 543.4 °C. The last liquid completely solidified at a temperature of 450.7 °C, which was very close to the final temperatures observed for the other alloys. The solidification start temperature was predicted to be 10.4 K smaller, from 644.5 to 634.1 °C, as compared to that of the Al-3%–Mg-0.1%–Sc alloy, with a slight decrease also in the start temperature of precipitation of the Al_3_Sc phase. The solidification range was approximately 184 K under nonequilibrium conditions, while under equilibrium conditions it was about 60 K.

In the case of the Al-10%–Mg-0.1%–Sc alloy, the solidification sequence depicted in Figure 2(c) exhibits some differences compared with that of the Al-5%–Mg-0.1%–Sc alloy. These include a decrease in the solidification start temperature to 606.7 °C, which was almost 30 °C smaller than that of the Al-5%–Mg-0.1%–Sc alloy. Additionally, there was a change in the precipitation sequence, with the *Al*_3_*Sc* phase forming earlier. The sequence was described as follows:
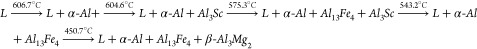
The solidification range was approximately 156 K under nonequilibrium conditions and around 97 K under equilibrium conditions. The same phases were predicted for all three alloys: *α-Al*, *Al*_3_*Sc*, *β-Al*_3_*Mg*_2_, and *Al*_13_*Fe*_4_. Coincidentally, the same phases were also predicted from the binary equilibrium diagrams. For all the examined alloys, there was an increase in the solidification range under nonequilibrium conditions compared to those of equilibrium conditions. Under nonequilibrium conditions, solute segregation is more likely to occur, allowing for phase precipitation at lower temperatures and decreasing the temperature at which the liquid phase exists.^30^

**Figure 3:**
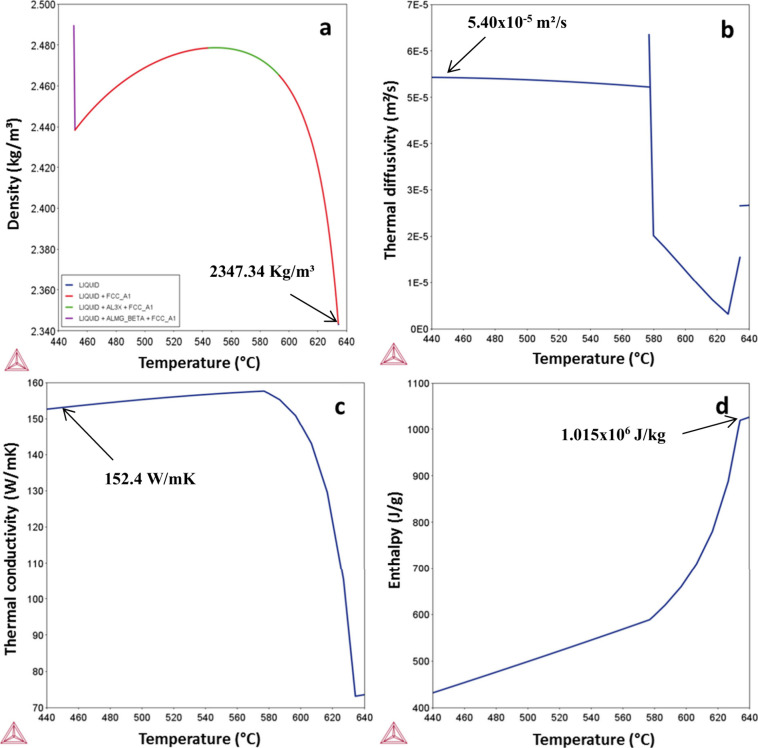
Calculations by the CALPHAD method demonstrate the determination of the thermophysical properties for the Al-5%–Mg-0.1%–Sc alloy.

The Thermo-Calc software was also used to determine key thermophysical properties of the Al–Mg–Sc alloys such as thermal diffusivity, thermal conductivity, enthalpy of melting, and density. Figure 3 depicts some of the plots generated by using Thermo-Calc considering the Al-5%–Mg-0.1%–Sc alloy. These properties were very useful to allow the application of the heat flow- and enthalpy-based models for predicting molten depth and width. A summary of the properties can be seen in Table 2.

**Table 2:** Laser Parameters (Track 1, Track 2, and Track 3), Experimental Molten Pool Depth and Width, and Thermophysical Properties (Extracted from Thermo-Calc) of the Al–Mg–Sc alloys

Alloy	Track	Power, *P* (W)	Speed, *V* (m/s)	Exp. depth, *D* (μm)	Exp. width, *W* (μm)	Liquid density (kg/m^3^)	Thermal conductivity (W/mK)	Thermal diffusivity (m^2^/s)	Enthalpy of melting (J/kg)
Al-3%–Mg-0.1%–Sc	1	250	0.025	78	230	2360.65	172.40	6.06 × 10^–5^	1.033 × 10^6^
	2		0.05	80	228				
	3		0.1	78	226				
Al-5%–Mg-0.1%–Sc	1	250	0.025	96	270	2347.34	152.41	5.40 × 10^–5^	1.015 × 10^6^
	2		0.05	93	257				
	3		0.1	87	253				
Al-10%–Mg-0.1%–Sc	1	250	0.025	124	313	2314.00	120.33	4.34 × 10^–5^	9.673 × 10^5^
	2		0.05	125	303				
	3		0.1	121	291				

**Figure 4:**
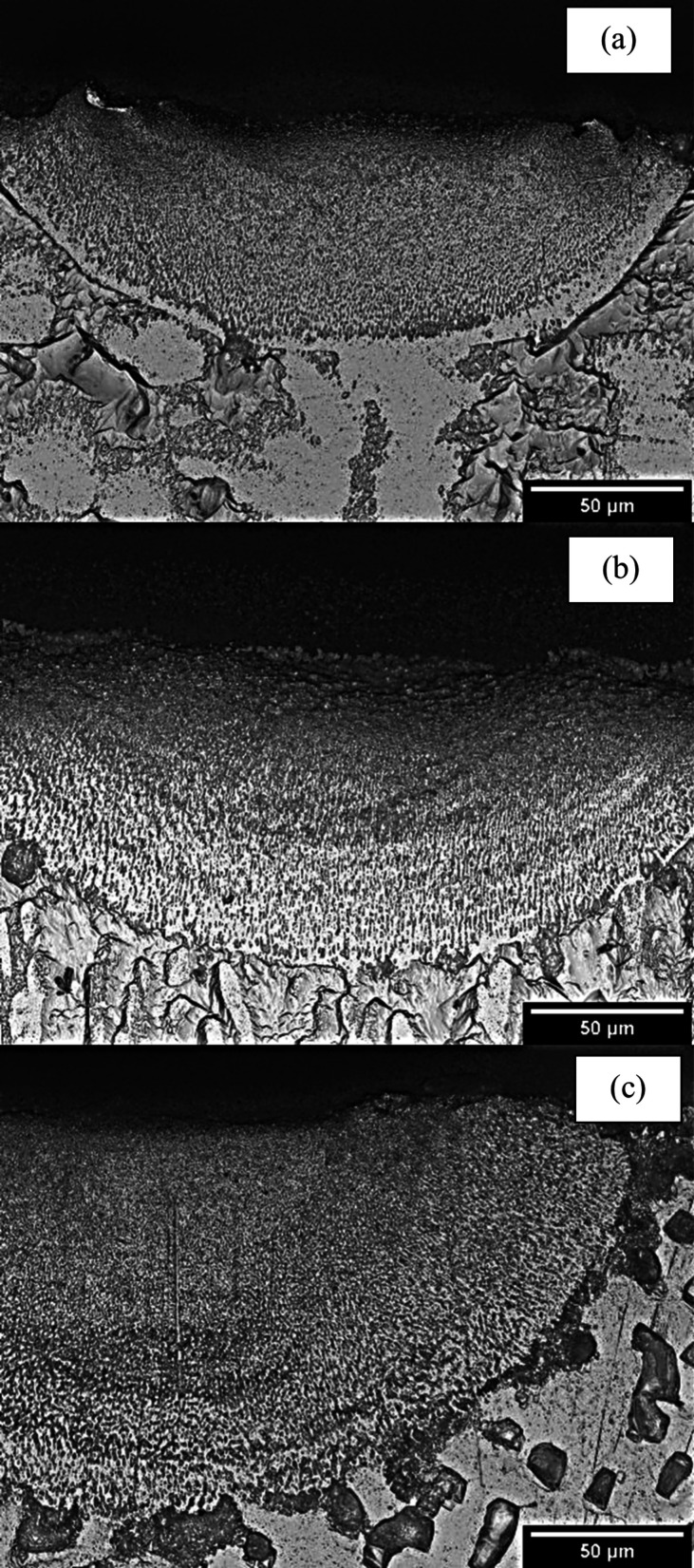
General views of the molten pools obtained after laser remelting with 10 J/mm (Track 1) in the (a) Al-3%–Mg-0.1%–Sc, (b) Al-5%–Mg-0.1%–Sc, and (c) Al-10%–Mg-0.1%–Sc alloys.

After performing metallography in all samples generated, important variations in the width and depth were noted experimentally. As can be seen in Figure 4 in the case of the Track 1, the molten pool size increased with the increase in the alloy Mg content. This was also the case for all of the other tracks. Table 2 also summarizes the width and depth values.

**Figure 5:**
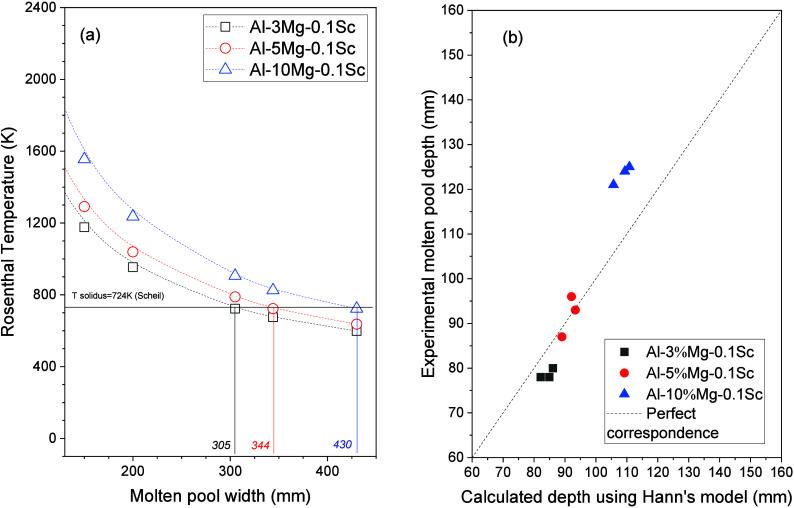
(a) Molten pool theoretical width temperature profiles obtained using Rosenthal’s equation considering remelted surfaces with 10 J/mm and (b) comparison between Hann theory and experimental measurements for the Al–Mg–Sc alloys.

The variation in size of the molten pool was explored by applying some theory, as can be seen in Figure 5. Both theories described qualitatively the same behavior as observed experimentally with the alloys, which means that they predicted increase in size as the alloy Mg content was increased. The Rosenthal equations allowed us to plot the temperature distribution of each alloy around the instantaneous heat source position in the plane of the width. The solidus isotherm determined through CALPHAD is represented by a horizontal dashed line in Figure 5(a), and it was considered as being the limit of the pool to be associated with the experimental width values. By comparing Figure 5(a) and Table 2 for 10 J/mm, the Rosenthal equations predicted width values of 305 mm, 344 mm, and 430 mm, exhibiting percentage errors of approximately 30% to 40% compared to the measurements in the alloys. These large errors can be attributed to the fact that this simplified model does not take into account heat transfer modes such as radiation and convection.^31^ This could also be a result of assumptions with which heat losses as well as latent heat of the alloy are supposed to be neglected. However, qualitatively, there is agreement with the experimental results. Hekmatjou et al.^31^ also demonstrated that the Rosenthal equation underestimated the thermal results of a AA5456 alloy processed with a pulsed Nd:YAG laser.

**Figure 6:**
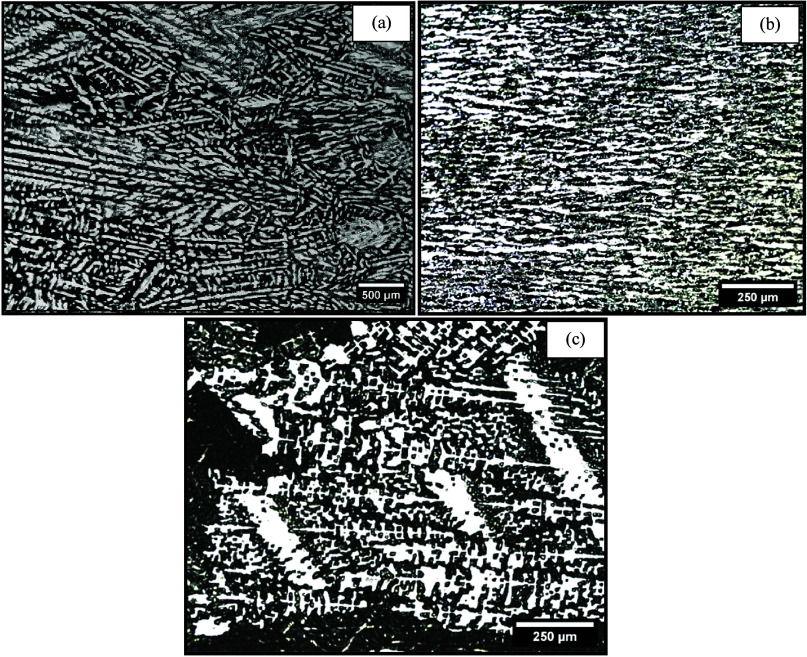
Typical microstructures of the cast plates (substrates) used as the basis for remelting processing of the (a) Al-3%–Mg-0.1%–Sc, (b) Al-5%–Mg-0.1%–Sc, and (c) Al-10%–Mg-0.1%–Sc alloys.

On the other hand, the Hann model presented predictions closer to the experimental values as compared to those demonstrated for Rosenthal approach (Figure 5(b)). A *C* constant of 0.017 was adopted for all calculations with the Hann model. While smaller errors (below ±8%) can be recognized for the Al-3%–Mg-0.1%–Sc and Al-5%–Mg-0.1%–Sc alloys, errors of approximately −12% and −13% were found in the analysis of the Al-10%–Mg-0.1%–Sc alloy data. Even considering Rosenthal, the results for the alloy with a higher Mg content were less accurate. This may be associated with the original microstructure from the casting process employed as a substrate. In the case of alloys with lower Mg content, coarse cells were revealed, whereas for the Al-10–Mg-0.1–Sc alloy, a very coarse dendritic microstructure was characterized, as shown in Figure 6. It appears that the coarser original structure of the as-cast plates could allow for higher thermal conduction,^32^ especially through the Al-rich phase, which occupies larger regions of the microstructure of the Al-10%–Mg–Sc alloy, thereby accelerating cooling and deviating further from the models. Vandersluis et al.^33^ demonstrated that the refinement of the spacing may reduce the number of mobility paths for conduction, decreasing the thermal conductivity.^33^ This kind of demonstration helps to justify the worst modeling results found for the Al-10%–Mg-0.1%–Sc alloy. The dendritic/cellular spacings of the Al-3%–Mg-0.1%–Sc alloy and Al-5%–Mg-0.1%–Sc substrate surfaces were on the order of 19 μm, whereas for the Al-10%–Mg-0.1%–Sc alloy, it was 35 μm. This coarsening may have influenced the worst modeling responses observed for the latter alloy.

The simple theory behind the Hann approach permitted a more accurate prediction of the change in size. This suggests that the molten depth is a function of the enthalpy of the alloy, which agrees with the physical interpretation of this problem. The enthalpy approach proposed by Hann et al.^34^ is similar in form to the Stefan number, which is used to predict the change in depth of a melt boundary in the classical Stefan’s problem.

**Figure 7:**
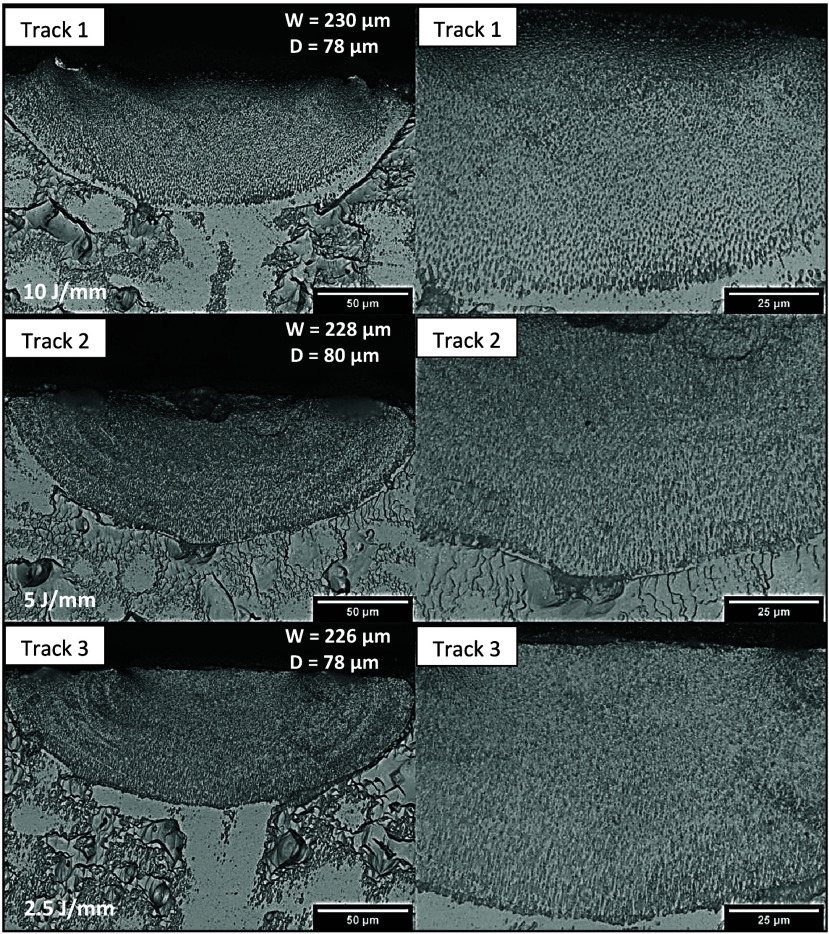
Optical microscopy images of the three tracks produced by LSR on the Al-3%–Mg-0.1%–Sc alloy: Track 1—25 mm/s, 250 W; track 2—50 mm/s, 250 W; track 3—100 mm/s, 250 W. The width (*W*) and depth (*D*) of the tracks are indicated in the lower magnification micrographs.

Highly refined microstructures can be observed in the micrographs of Tracks 1, 2, and 3 of the Al-3%–Mg–Sc alloy, as depicted in Figure 7. Cells can be observed at higher magnification (at the right side images).

**Figure 8:**
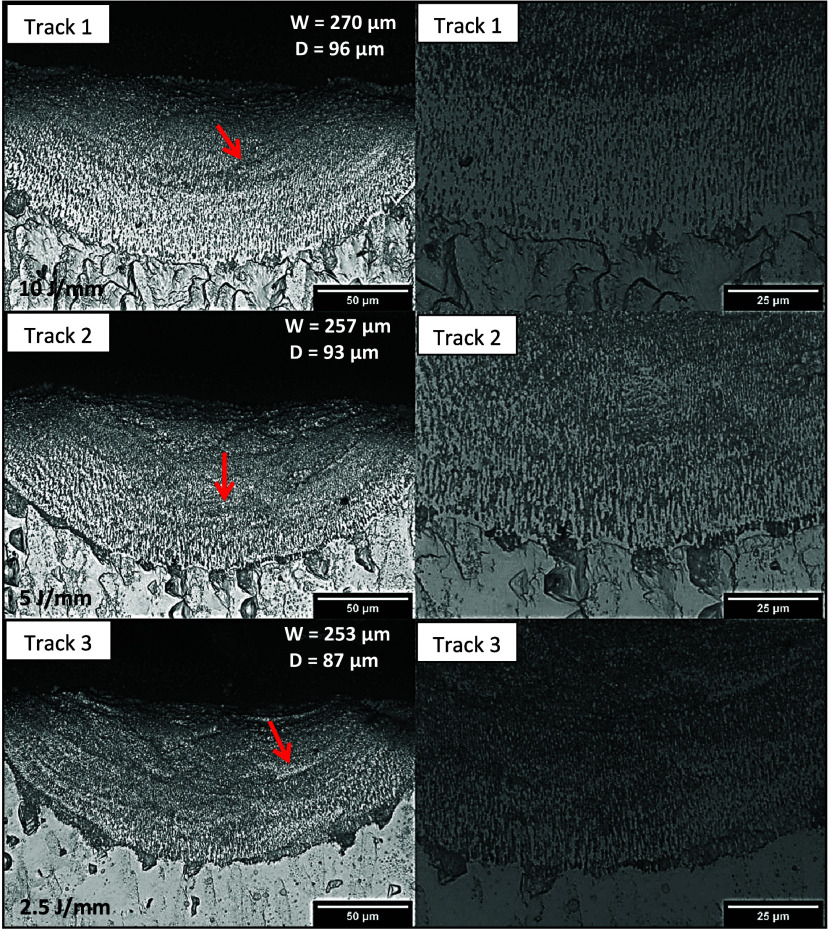
Optical microscopy images of the three tracks produced by LSR on the Al-5%–Mg-0.1%–Sc alloy: Track 1—25 mm/s, 250 W; track 2—50 mm/s, 250 W; track 3—100 mm/s, 250 W. The width (*W*) and depth (*D*) of the tracks are indicated in the lower magnification micrographs. [Adapted with permission from Laser resolidification of Al-5%–Mg-0.1%–Sc alloy: Growth of cells and banded structures, *Journal of Alloys and Compounds*, **2024**, *973*, 172889, 10.1016/j.jallcom.2023.172889. Copyright (2024) Elsevier.]

Similar aspects and cell growth were also observed for the Al-5%–Mg-0.1%–Sc alloy, as highlighted in Figure 8. Regions indicated by arrows point to possible localized compositional fluctuations (bands) within the tracks. In-depth microstructural analyses of band formation in laser processing have been carried out in previous studies^35,36^ and have revealed that these compositional fluctuations consist of a sequence of light and dark bands that develop approximately parallel to the solid–liquid interface. The dark bands have a dendritic or eutectic structure, while the light bands do not exhibit microsegregation and are probably the result of a flat front development morphology. Higher solidification rates appear to trigger the growth of biphasic bands in conjunction with cellular growth under the present condition. There was no defined or discernible transition between these structures.

The variation in width (*W*) and depth (*D*) may indicate the influence of heat input on the track dimensions for the remelted Al-5%–Mg-0.1%–Sc alloy. After measuring the track dimensions, it was observed that those obtained under conditions of higher heat input showed a tendency to increase in area, which implies that a larger volume of material was remelted due to the passage of the laser beam. Since the tracks in question were obtained with the LSR treatment using the same power of 250 W, when the laser beam displacement speed (*V*) over the substrate was slower, the heat input was greater, and therefore, the beam interacted for a longer time with the region of the remelted substrate, promoting an increase in the track dimensions, more noticeable with respect to the width.

**Figure 9:**
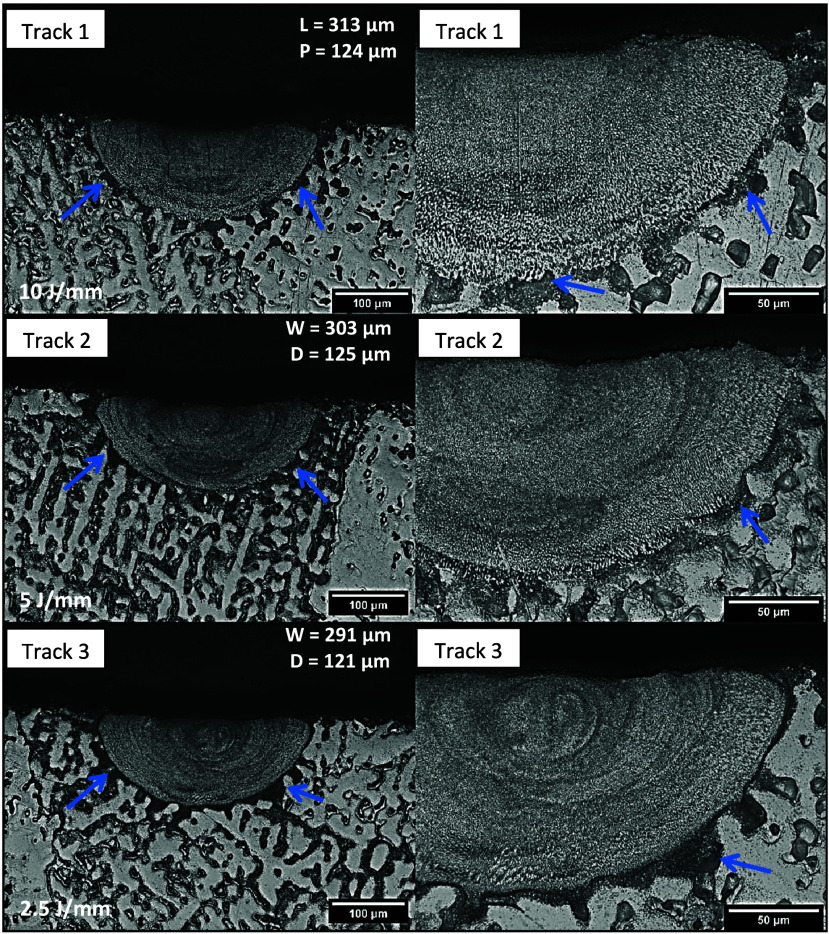
Optical microscopy images of the three tracks produced by LSR on the Al-10%–Mg-0.1%–Sc alloy: Track 1—25 mm/s, 250 W; track 2—50 mm/s, 250 W; track 3—100 mm/s, 250 W. The width (*W*) and depth (*D*) of the tracks are indicated in the lower magnification micrographs.

Refined cells can also be observed in the microstructures of the tracks in Figure 9, banded regions as in the previous tracks, and, more prominently, a more robust interface area (see arrows in the micrographs). This other important microstructural aspect observed in the micrographs is a probable product of interfacial remelting from the original solid (substrate). This occurs due to the passage of the laser beam, the formation of which influences the growth of cells and dendrites at the base of the molten pool. Consequently, the resulting molten pool tends to penetrate slightly into areas of the substrate where the liquid reaches equilibrium with the lower melting point eutectic microconstituent, as opposed to the regions where there is equilibrium between the α-Al (Mg) phase and the molten alloy. The influence of the substrate composition on the formation of the solid–liquid interface during laser-induced melting is evident in the resulting micromorphology. This results in the formation of an extremely thin layer of altered eutectic products that adopts a cellular growth pattern. Similar observations have been reported for Al–Cu alloys.^37,38^ Increased heat input may have contributed to increase in the width (*W*) and depth (*D*) of the tracks for the Al-10%–Mg-0.1%–Sc alloy since higher heat input means longer interaction time of the laser beam with the substrate.

The microstructural characteristics of the laser tracks of the Al–Mg–Sc alloys that were presented in the micrographs in Figure 7 to Figure 9 highlighted the presence of highly refined cells in all tracks, regions of compositional fluctuations, henceforth treated as banded or banding areas.^35^ Also, the influence of the Mg content on the formation of banding, increase of the interfacial region between the track and the substrate, and increase of the track dimensions can be evaluated. For alloys with a higher Mg content, the heat input indicated a directly proportional relationship with the track dimensions because of the longer interaction time of the laser with the substrate area.

Table 3 presents qualitative estimations of the cellular, intercellular, and banded fractions as well as the cellular spacing corresponding to the bottom molten pools obtained by LSR with the Al–Mg–Sc alloys.

**Table 3:** Summary of Cellular, Intercellular, Banded Fractions, and Cellular Spacing Related to the Tracks Obtained by LSR at a Laser Power of 250 W in the Laser Remelted Al–Mg–Sc Alloys

Alloy	Cellular spacing of the substrate surface (μm)	Heat input (J/mm)	Cellular spacing at the pool bottom (μm)	Cellular fraction	Intercellular fraction	Banded fraction
Al-3%–Mg-0.1%–Sc	19.1	Tracks 1–10	1.20	0.56	0.24	0.20
		Tracks 2–5	1.02	0.58	0.30	0.12
		Tracks 3–2.5	0.88	0.56	0.28	0.16
Al-5%–Mg-0.1%–Sc	19.8	Tracks 1–10	1.20	0.65	0.32	0.03
		Tracks 2–5	1.11	0.52	0.29	0.18
		Tracks 3–2.5	0.97	0.36	0.20	0.44
Al-10%–Mg-0.1%–Sc	35.4	Tracks 1–10	1.56	0.42	0.24	0.34
		Tracks 2–5	1.14	0.41	0.24	0.35
		Tracks 3–2.5	0.74	0.49	0.25	0.27

Table 3 indicates that for all of the studied Al–Mg–Sc alloys, the cellular spacing (λ_*C*_) characterizing the tracks is proportional to the heat input. In absolute values, there are spacings as small as 0.74 μm up to higher values such as 1.56 μm. The smallest cellular spacing values were observed for the Al-10%–Mg-0.1%–Sc alloy. On average, λ_*C*_ is in the range from 1.03 to 1.15 μm in the tracks. Comparing the microstructural spacing of the substrates with the λ_*C*_ of the cells inside the tracks, λ_*C*_ varied between 16× (track 1, Al-3%–Mg-0.1%–Sc) and 48× (track 3, Al-10%–Mg-0.1%–Sc) smaller. The highest microstructural refinement with respect to the substrate occurred for the Al-10%–Mg-0.1%–Sc alloys. Regarding the fractions, while the average intercellular fraction considering each alloy oscillates between 0.24 and 0.27, the average cellular fraction is between 0.44 and 0.57, and the average banded fraction varies from 0.16 to 0.32. Observing the tracks individually for a single alloy, there seems to be an inversely proportional relationship between the cellular fraction and the banded fraction, since the intercellular fraction showed little variation.

Analysis of Table 3 does not clearly reveal a correlation between the alloy Mg content and the cellular and banded fractions. However, when the micrographs are observed, the banded fraction appears to increase with an increasing Mg content in the alloy composition. To provide further evidence and clarify a possible correlation between the Mg content and the banded fraction of the tracks, point MEV/EDS analyses were carried out in distinct regions of some tracks.

**Figure 10:**
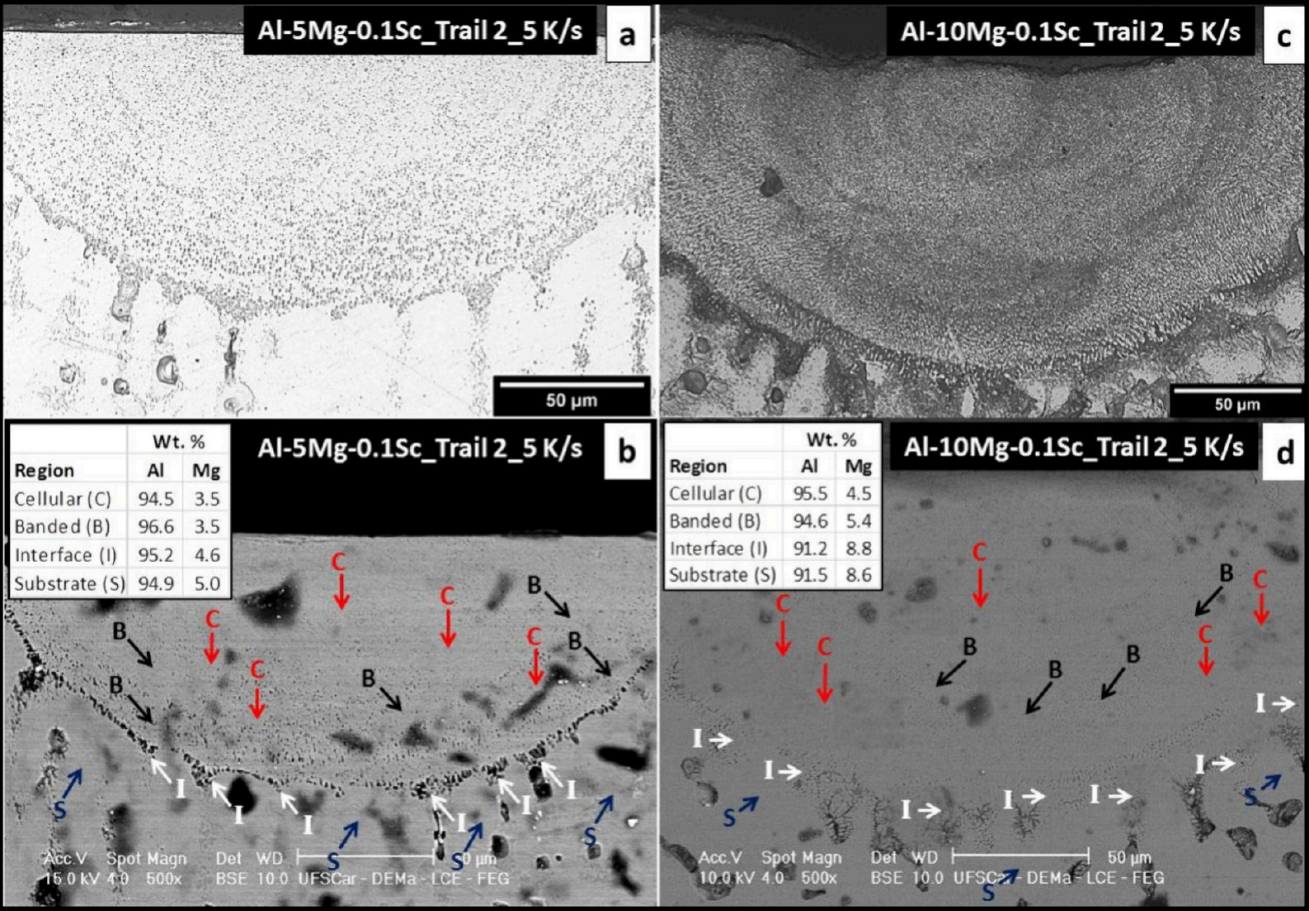
Optical micrograph and SEM images corresponding to the same regions of track 2 of LSRed samples produced with the (a, b) Al-5%–Mg-0.1%–Sc and (c, d) Al–10%–Mg-0.1%–Sc alloys. Boxes on the (c) and (d) images indicate the average percent composition, measured by punctual EDS analyses on the cellular regions (C), banded structures (B), at the intercellular (I), and on the substrate (S).

Figure 10 shows the combination of optical micrographs and SEM images in both Al-5%–Mg-0.1%–Sc and Al-10%–Mg-0.1%–Sc alloys considering Track 2. Banded regions are more noticeable in the Al-10%–Mg-0.1%–Sc alloy. In the SEM images, “B” denotes banded regions, “C” represents cellular regions, “I” indicates intercellular regions, and “S” stands for the substrate. The corresponding average weight percentages of Al and Mg are presented in the inset boxes in Figure 10. Points “B”, “C”, “I”, and “S” also mark the locations chosen for the EDS analysis. While the alloy nominal content was maintained in the intercellular region, both the cellular and banded regions exhibited some Mg depletion. Regarding the cellular region, this Mg depletion can be attributed to solidification conditions induced by the LSR process. In the case of the banded region, this may have resulted from the selective etching during metallographic preparation. The choice of the etchant was centered on its ability to reveal cellular regions, but due to selectivity of the reaction, Mg in the intercellular and banded regions formed after the LSR process was selectively eliminated.

**Figure 11:**
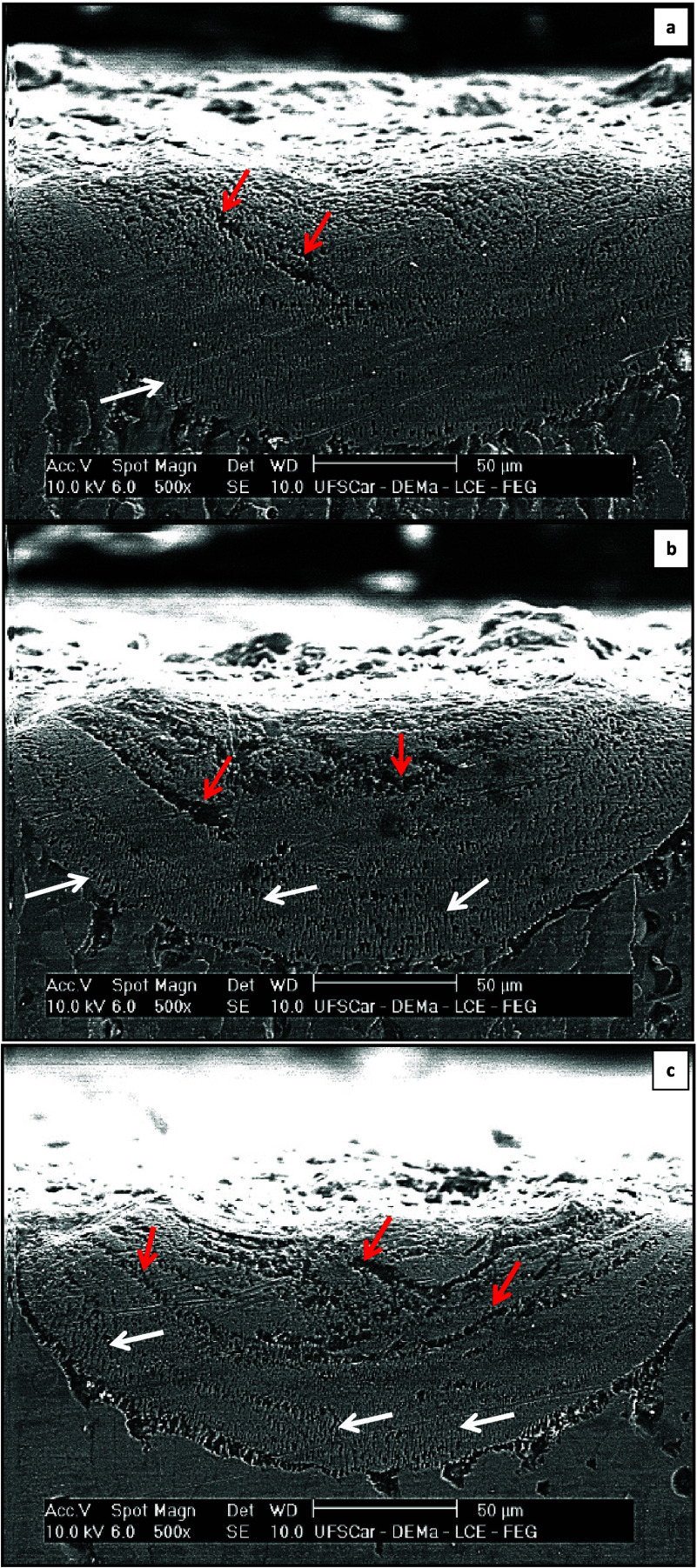
SEM images of three laser remelted samples on the surface of the Al-5%–Mg-0.1%–Sc alloy substrate: (a) Track 1—10 J/mm, (b) Track 2—5 J/mm, and (c) Track 3—2.5 J/mm. [Adapted with permission from Laser resolidification of Al-5%–Mg-0.1%–Sc alloy: Growth of cells and banded structures, *Journal of Alloys and Compounds*, **2024**, *973*, 172889, 10.1016/j.jallcom.2023.172889. Copyright (2024) Elsevier].

The SEM images in Figure 11 allow the morphologies to be identified and discussed more clearly. It can be observed that the formation of a microstructure is composed of bands (red arrows) and alternating cells (white arrows) in the Al–Mg–Sc alloy, especially at higher cooling rates, or in other words, at smaller heat input, at a power of 250 W for all the tracks generated (see Figure 11(c)).

This type of microstructural alternation has already been reported by Zimmermann et al.,^35^ who demonstrated that, in the high-speed regime (cell growth here), the velocity front can instantaneously increase to a new level of even higher speeds (bands). At this point, the structure is growing too rapidly relative to the advancing isotherm and, therefore, will decelerate, returning to the original cell velocity regime. The result is the formation of a banded structure. Another notable aspect is the change in the growth orientation of the cells near the upper section of the molten pool, a phenomenon documented similarly in the cases of Al–Cu and Al–Ni alloys.^39,40^ At the periphery of the track, the growth direction is perpendicular to the scanning path of the laser beam, making a progressive transition to a parallel alignment as it reaches the upper surface.

**Figure 12:**
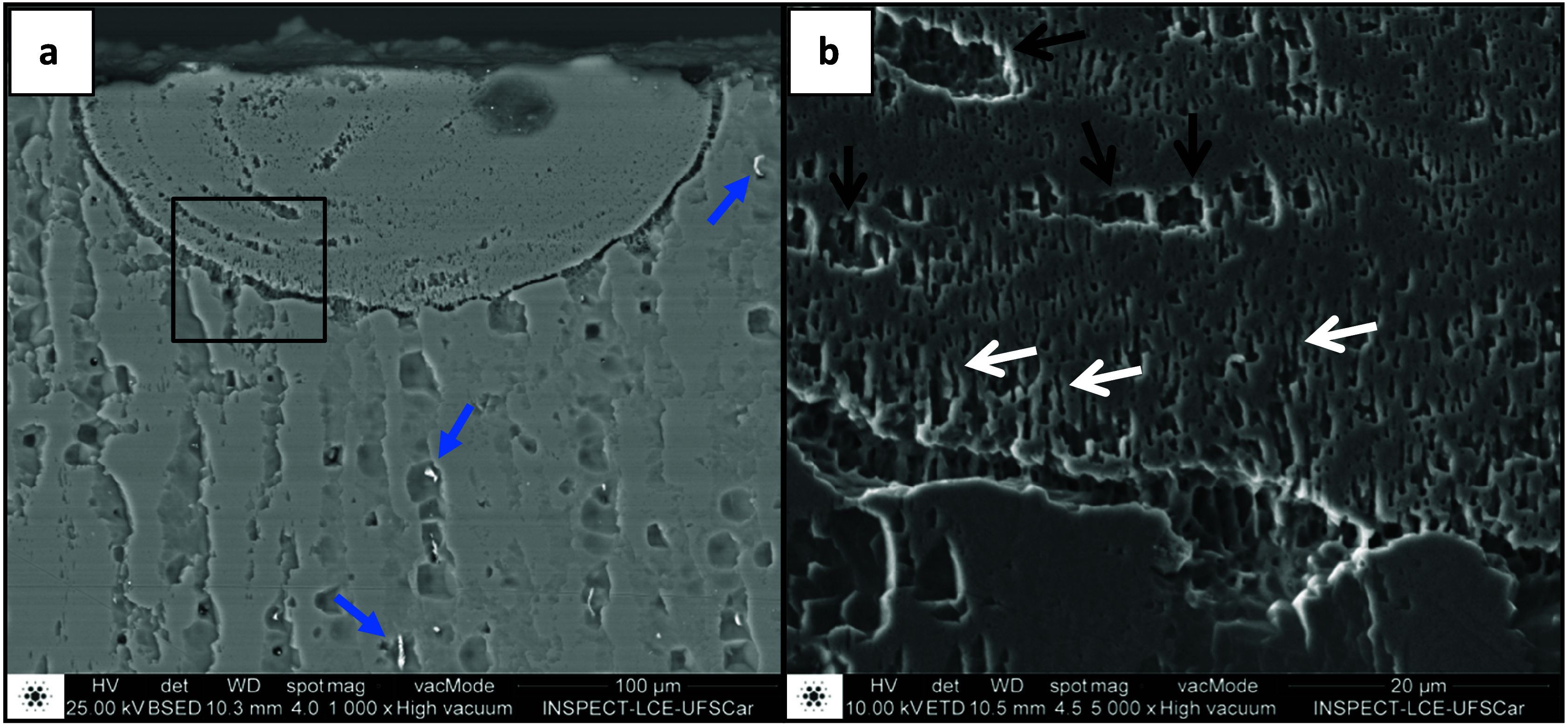
SEM image of the laser remelted Track 3 (2.5 J/mm) on the surface of the Al-5%–Mg-0.1%–Sc alloy substrate. In (b), cells and banded regions can be observed at 5000× magnification of the region in the detail highlighted in (a).

Figure 12 shows SEM images of track 3 in the Al-5%–Mg-0.1%–Sc alloy. Figure 12 (b) is a magnification of the region detailed in (a), in which the black arrows indicate banded regions, while the white arrows point to the cells. In the lower magnification image, brighter particles can be observed that correspond to the AlFe phase (indicated by blue arrows) in the intercellular regions of the substrate. These particles are due to the Fe impurity in the alloy to produce the plates and are not observed inside the laser tracks. The equilibrium solubility of Fe in Al is only 0.05 wt %, while under metastable conditions the solubility can reach up to 8 wt % under high cooling rates such as those observed in laser remelting operations.^41^ This difference explains the non-observation of Fe-containing phases in the laser-treated zone.

### 3.2 Microstructural Coarsening and Hardness

**Figure 13:**
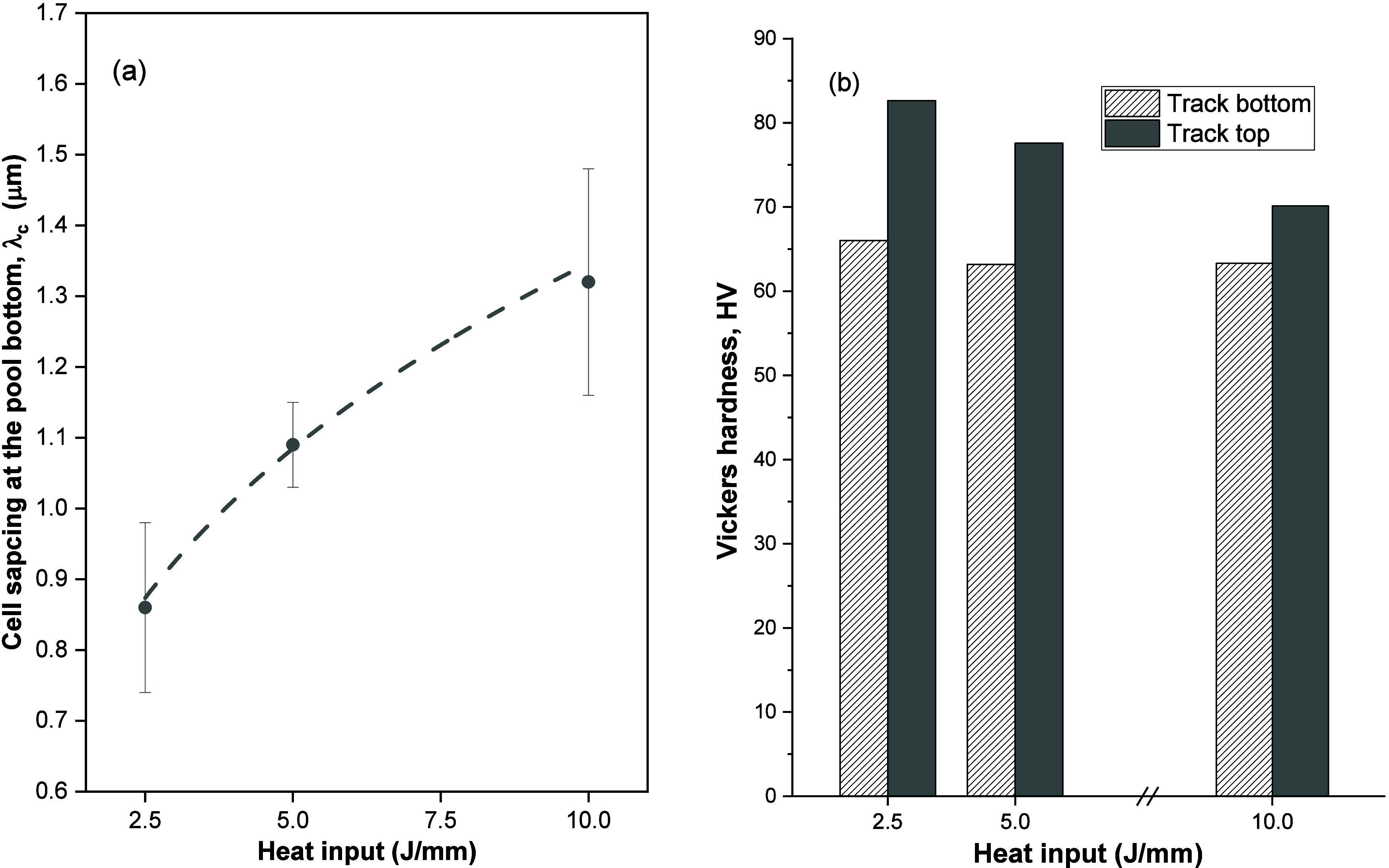
(a) Cell spacing at the molten pool bottom of the Al–Mg–Sc alloys changes as a function of the heat input and (b) hardness variation as a function of heat input at the bottom and at the top of the tracks.

As the 3 (three) examined alloys do not show large differences in microstructural spacing among them, the spacing data was put together as a function of the heat input. As such, the influence of heat input on the average microstructural spacing at the track bottom part, combining the 3 alloys studied, can be observed in Figure 13(a). The higher the heat input, the higher the λ_*C*_, which may have an influence on the Vickers microhardness of the laser-treated region.

It was also important to follow differences in hardness from the bottom to the top of the laser tracks. As such, the same approach was carried out by combining hardness data of the 3 alloys as can be seen in Figure 13(b). The graphs clearly indicate that the microhardness increases with decreasing heat input, especially at the top of the treated regions.

**Figure 14:**
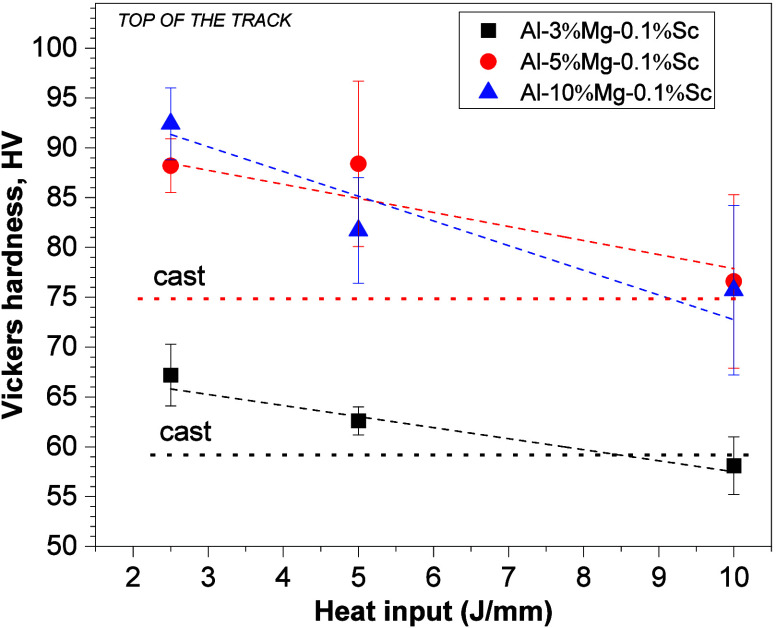
Average Vickers microhardness versus heat input corresponding to the top of the laser-modified regions referring to the Al-(3, 5, and 10)%–Mg-0.1%–Sc alloys.

Figure 14 shows the hardness measurements on the top of the tracks as a function of the heat input of the LSR. From the curves plotted from the experimental points, it can be observed that the Al-3%–Mg-0.1%–Sc and Al-5%–Mg-0.1%–Sc alloys showed a tendency for hardness to increase with decreasing heat input, with hardness values equal to those of the as-cast substrates when considered the highest measured heat input, i.e., 10 J/mm. On the other hand, the Al-10%–Mg-0.1%–Sc alloy showed a tendency for hardness to increase with decreasing heat input, but the hardness was lower than the average as-cast substrate, which attained 109 HV when slowly solidified between 2 and 5 K/s. Moreover, it seems that high solidification rates and more refined structures at the top of the tracks tended to equalize the hardness evolution for alloys with a higher Mg content.

**Figure 15:**
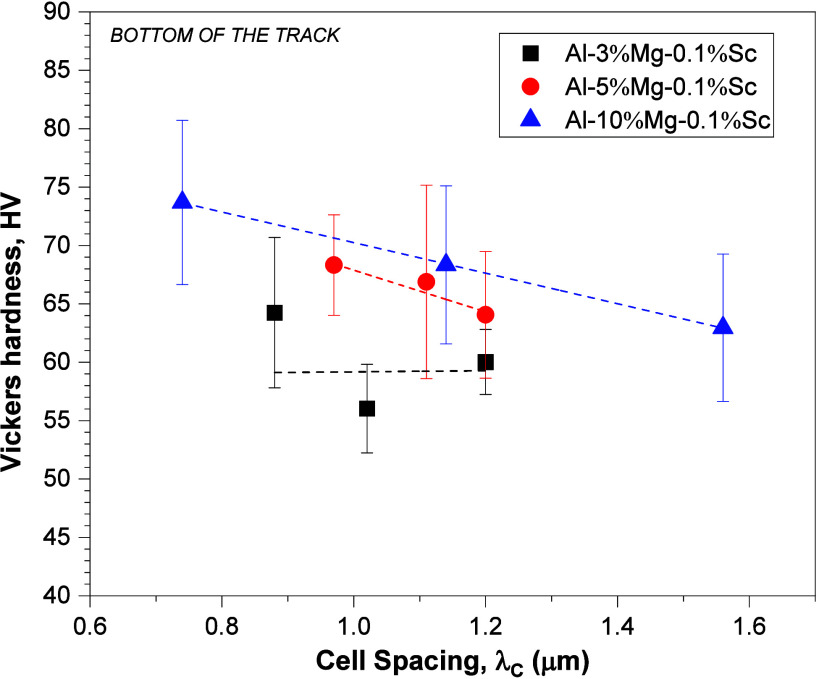
Correlations of Vickers microhardness versus cell spacing corresponding to the molten pool bottom of the laser-modified regions in the Al-(3, 5, and 10)%–Mg-0.1%–Sc alloys.

Regarding the hardness of the base of the laser-treated region of the LSR-treated samples, Figure 15 shows the Vickers microhardness measurements as a function of λ_*C*_. These analyses were performed in the bottom regions of the molten pool, as the values of λ_*C*_ were mapped in these regions. It is observed that the Al-5%–Mg-0.1%–Sc and Al-10%–Mg-0.1%–Sc alloys showed a clear tendency for an increase in microhardness with decreasing λ_*C*_. Although no suitable correlation was found between the microhardness and λ_C_ for the Al-3%–Mg-0.1%–Sc alloy, the microhardness corresponding to the lowest value of λ_*C*_ increased by approximately 10% as compared to that of the corresponding as-cast sample. The Al-10%–Mg-0.1%–Sc alloy samples showed the highest hardness among all three alloys examined. Higher Mg content is found in both the bands and the cells as observed in Figure 10, which corroborates to higher hardness in this case.

The magnitude of the microhardness of the Al-5%–Mg-0.1%–Sc alloy was lower than that of the as-cast sample, however, and according to the trend indicated by the curve, a decrease in λ_*C*_ to critical values close to 0.6 μm would contribute to an increase in the microhardness in the remelted region to similar average microhardness levels of approximately 76 HV. This observed reduction in the magnitude of the microhardness of the laser-remelted Al-5%–Mg-0.1%–Sc alloy may have been caused by microstructural changes such as the reduction of interdendritic/intercellular areas. To support this hypothesis, the interdendritic/intercellular areas of the as-cast substrate samples were measured using the same methodology employed for estimating the cellular, intercellular, and banded fractions in the remelted tracks. For the as-cast substrate, the fraction corresponding to the intercellular regions was 54%. Comparing these results with the fractions estimated for the intercellular regions in the remelted pools (Table 3), LSR caused a reduction in the intercellular fraction, whose fraction was estimated to be around 25%.

Considering an extrapolation of the curves in Figure 15 there seems to be a critical cellular spacing value that would need to be achieved for there to be a gain in hardness through LSR. Some process window possibilities involve decreasing the heat input, which can be done by reducing the laser power or increasing the laser scanning speed. In addition to saving energy, decreasing the power minimizes the possibility of typical welding defects, such as cracks and pores or even the occurrence of the keyhole phenomenon. On the other hand, increasing V reduces processing time, increasing process productivity.

Ignoring the depth of the molten pool, in economic terms, decreasing the heat input seems to be attractive for improving the hardness, especially in the Al-5%–Mg-0.1%–Sc and Al-10%–Mg-0.1%–Sc alloys. It should also be considered that as discussed earlier in the SEM results, LSR promoted the dissolution of precipitates present in the original substrate. Therefore, under high heat input conditions, the lower hardness may be attributed to the absence of hardening precipitates. The higher hardness at lower heat input may be a combination of the microstructural refinement of the formed cells and some fraction of the remaining precipitates.

## 4 Conclusions

Key thermophysical properties of the Al–Mg–Sc alloys, such as thermal diffusivity, thermal conductivity, enthalpy of melting, and density, were determined by the Thermo-Calc software, thus allowing the application of heat flow and enthalpy-based models for predicting depth and width of the laser molten pools. The experimental molten pool size was shown to increase with the increase in the alloy Mg content, which was qualitatively modeled. The Hann’s model was more precise in determining the track depths. According to Hann et al.^21^ this simple model, while not as potentially accurate as numerical modeling, provides sufficient detail to explain many key features of the track geometry. It is easy to apply and would be of greater interest for industrial applications, where there is limited capability to run complex simulations. Moreover, the normalized enthalpy used in this model is similar in form to the Stefan number, which predicts the change in the depth of a melt boundary in the classical Stefan problem.

The microstructure of the laser tracks was characterized by the presence of highly refined cells in all tracks, regions of compositional fluctuations, and henceforth treated as banded areas. The cellular spacing (λ_*C*_) varied from 0.74 μm up to values as high as 1.56 μm. The smallest λ_*C*_ values were observed for the Al-10%–Mg-0.1%–Sc alloy. Moreover, this alloy resulted in higher fractions of bands forming laser-treated areas.

The hardness at the top of the track was higher than that at the bottom due to the inherent higher solidification velocities at the top. The Al-5%–Mg-0.1%–Sc and Al-10%–Mg-0.1%–Sc alloys showed a clear tendency for an increase in microhardness with decreasing λ_*C*_. Although no suitable correlation was found between the microhardness and λ_*C*_ for the Al-3%–Mg-0.1%–Sc alloy, the microhardness corresponding to the lowest value of λ_*C*_ increased by approximately 10% as compared to that of the corresponding as-cast sample. The Al-10%–Mg-0.1%–Sc alloy samples showed highest hardness among all the three alloys examined. The current laser processing conditions did not enhance the hardness at the bottom of the tracks. Attaining a critical cellular spacing value appears necessary for hardness improvement via LSR. For that, potential process adjustments include either lowering the heat input or increasing the laser scanning speed.
